# Social Isolation and Hospitalization Risk Factors Among Community-Dwelling Older Adults

**DOI:** 10.1093/geroni/igaa057.314

**Published:** 2020-12-16

**Authors:** J Mary Louise Pomeroy, Gilbert Gimm

**Affiliations:** George Mason University, Fairfax, Virginia, United States

## Abstract

PURPOSE: This study examines psychosocial risk factors associated with hospitalization among community-dwelling older adults in the United States. METHODS: Using two waves of the National Health and Aging Trends Study from 2011 and 2015, we conducted descriptive and multivariate analyses of individual-level data from a nationally representative sample of 8,003 Medicare beneficiaries ages 65 and older. Associations between hospitalization and risk factors including social isolation, depression, and anxiety were assessed. Covariates included gender, race/ethnicity, age, region, insurance type, falls, and comorbidities. RESULTS: Overall, about 20.9% of older adults reported a hospitalization within the past year and 22.2% were socially isolated. The odds of hospitalization were higher for socially isolated adults (OR 1.17; p = .02), for depressed adults (OR 1.25; p = .01), and for individuals with anxiety (OR 1.25; p = .02). Individuals living in the Western region had lower odds of hospitalization (OR 0.71; p = .001), whereas men (OR 1.13; p = .03), those requiring assistance with activities of daily living (OR 1.48; p < .001), and those having one (OR 1.41; p = .03) or more (OR 3.05; p < .001) chronic health conditions had higher odds of hospitalization. CONCLUSION: Social isolation, depression, and anxiety represent significant psychosocial risk factors for hospitalization among community-dwelling older adults in the United States. Efforts to reduce health care costs and improve health outcomes for older adults should explore ways to strengthen social integration and improve mental health.

